# Supplementary material for: Whole Exome Sequencing Identifies PHF14 Mutations in Neurocytoma and Predicts Responsivity to the PDGFR Inhibitor Sunitinib
Source: Biomedicines. 2022 Nov 8;10(11):2842. doi: 10.3390/biomedicines10112842 (PMC9687778; doi:10.3390/biomedicines10112842)
Supplement: Supplementary file 1 [file biomedicines-10-02842-s001.zip › biomedicines-1920735-Figure S1.pdf]

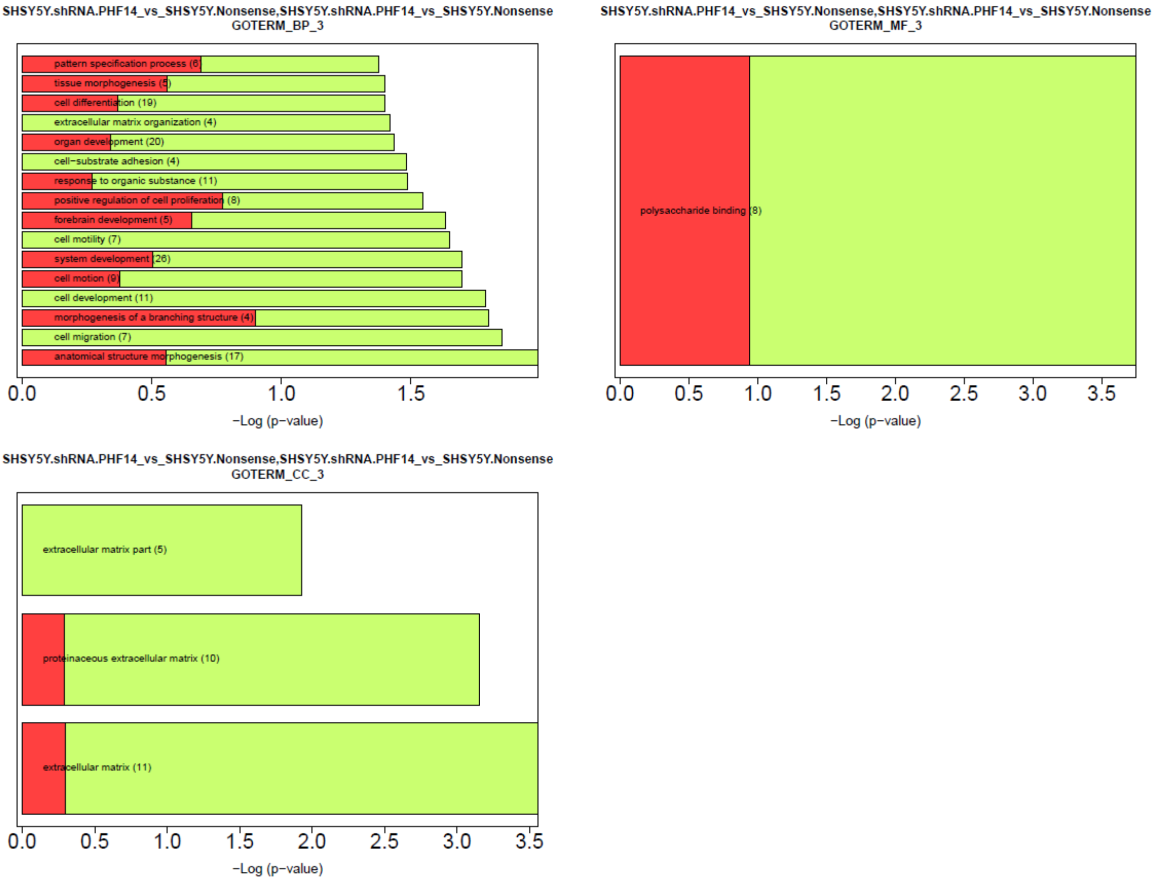

Figure S1. Gene Ontology analysis of mRNA profiles in SHSY-5Y nonsense control cells and shRNA PHF14 knockdown cells was performed to characterize changes in biological process (BP), molecular function (MF) and cellular components (CC) caused by PHF14 knockdown.
